# Publisher Correction: Invisible Trojan-horse attack

**DOI:** 10.1038/s41598-017-14859-y

**Published:** 2017-11-22

**Authors:** Shihan Sajeed, Carter Minshull, Nitin Jain, Vadim Makarov

**Affiliations:** 1 Institute for Quantum Computing, University of Waterloo, Waterloo, ON N2L 3G1 Canada; 2 Department of Electrical and Computer Engineering, University of Waterloo, Waterloo, ON N2L 3G1 Canada; 3 Department of Physics and Astronomy, University of Waterloo, Waterloo, ON N2L 3G1 Canada; 4 Department of Physics, Technical University of Denmark, Fysikvej, Kongens Lyngby 2800 Denmark

*Scientific Reports* **7**:8403; doi:10.1038/s41598-017-08279-1; Article published online 21 August 2017

In the original version of this Article, the Executive Summary section was omitted.

This error has now been corrected in the PDF and HTML versions of the Article.

